# Proceedings of the Society of Electro-Therapeutists

**Published:** 1896-01

**Authors:** Clara E. Gary

**Affiliations:** Secretary


### PROCEEDINGS OF THE SOCIETY OF ELECTRO-THERAPEUTISTS.

The third annual meeting of the National Society of Electro-Therupeutists was held at Hotel Vendome, September 18th and 19th, 1895.

Secretary Dr. Clara E. Gary reported an increase of twenty-five members during the past year. Treasurer J. B. Garrison, M. D., reported the finances of the Society to be in a good condition.

The President, Dr. William L. Jackson, then gave the annual address upon " The Development of Electro-Therapeutics and its relation to the Practice of Medicine." He advised all physicians to investigate the subject and satisfy themselves of the value of electricity as a therapeutic agent. There are clearly-defined indications for its use, and it has a sphere of usefulness all its own, which must be recognized. A number of interesting papers followed. Among them several showed original investigation in new fields.

The first paper read was that on "Some Experiments on Cataphoresis and Anaphoresis," by W. H. King, M. D., and F. M. Frazer, M. D., of New York. The authors divided Electrical Osmosis into Cataphoresis and Anaphoresis, and stated that their experiments proved conclusively that the positive and not the negative pole attracts Iodine, a result in direct opposition to the previous belief.

"Catalytic Effects of the Galvanic Current" was a paper read by Charles Porter Hart, M. D., of Wyoming, Ohio, in which he stated that his practice was to place the cathode upon the seat of the trouble in acute and inacute cases, while in chronic cases, especially if complicated with paralysis, he used the anode at the seat of the diseases.

In a paper entitled "Is the Practice of Electro-Therapeutics a Fad Practice?" E. S. Bailey, M. D., of Chicago, Ill., says that the results obtained in private practice had convinced him of the efficacy of electricity, and it is those who do not understand it who call it a fad.

"Electricity in Dyspepsia," by Dr. M. T. G. Lajoie, of New York, was the next paper. The Doctor found great benefit from the use of static electricity in dyspepsia, especially those cases complicated with neurasthenia.

Mr. J. Emory Clapp gave a review of "The First Principles of Electricity."

Mr. T. F. Livingston, of New York, discussed "The Difficulties of Utilizing Commercial Currents for Therapeutic Purposes from an Electrician's Standpoint," and showed the undesirability of the use of the street current in ordinary electro-therapeutical work.

In "Treatment of Rheumatism by Electricity," Chester G.

Higbee, M. D., of St. Paul, Minn., gave as his experience that he had found benefit from the use of electricity in rheumatism, using the indicated remedy under the positive electrode applied to the diseased parts.

The next paper was entitled "The General Therapeutic Effect of the Alternating Electric Current of High Frequency and of High Tension," by Dr. G. Apostoli, of Paris, France. The Doctor found it beneficial in these cases where there is defective nutrition, particularly in rheumatism and gout.

The paper written by William Harvey King, M. D., of New York, on "Locomotor Ataxia" was very interesting. He made the point that failure in the treatment of locomotor ataxia is largely due to an improper selection of the kind of current in different stages of the disease. He finds in the first stage more benefit from the galvanic current. Static electricity holds a prominent place in the treatment in the later stages. The case in which it is indicated should be carefully chosen, and promises should not be given regarding its permanent effect.

Following this paper came "A Review of a Case of Bell's Paralysis," by Julia Gould Waylan, M. D., of Philadelphia, Pa.

Then came a paper by N. B. Delemater, M.D., of Chicago, Ill., on "The Selection of the Current in the Treatment of Neuritis." In cases of chronic neuritis, the inflammation having subsided while malnutrition and partially paralyzed muscles are still existing as the result of the neuritis, the Faradic current should be used, as it stimulates nutrition, restores natural contractility, and overcomes abnormal contractions.

"The Mitigating Effect of Spinal Galvanism upon the Subjective Symptoms of Tabes," by Frank C. Richardson, M. D., of Boston, reported very good results from the use of the ascending current from 15 to 25 M. A., making the anode active over every part of the spine, moving up.

"Do Electrical Currents Affect the Spinal Ganglia?" by E. P. Colby, M. D., of Boston, brought out considerable discussion upon the above question, showing a diversity of opinion, and yet it was generally believed that a certain portion of the current would find its way to the spinal cord itself.

F. M. Frazer, M. D., of New York, in his paper entitled "Faradic Polarity," showed that the recent belief that there was no difference in the poles has been entirely disproved, and that the break-current predominates. It does have a slight polar effect, as was shown by its action on Iodide of Potash and Starch.

The evening session opened with a paper by J. Inglis Parsons, M. D., M. R. C. P., of London, England, on "The Disintegration of Organic Tissues by High-Tension Currents." Dr. Parsons showed that it is possible to cause destruction of tissue by means of rapid alternations of the high voltage current, but of small amperage. This does not cause sloughing, but is followed by atrophy of the tissue treated.

Professor William L. Puffer, of Boston, in his paper on "Polyphased Currents and Their Effects," said that the polyphased currents are only produced by a dynamo at the same time, only out of step, as it is called, or at different periods from each other.

"Polaphased Currents," by Professor Edwin J. Houston and E. A. Kennelly, of Philadelphia, Pa., maintained that from a physical point of view it is not possible that any peculiar physiological effect can be expected by the use of multiphase currents in contra-distinction to ordinary uniphase currents. If the existence of such effect can be demonstrated, it will be dependent upon the existence of some time relationship between the development of the current waves in regions where they cross each other's paths at nearly right angles.

"Electro-magnetic Basis of Physiology," by Professor A. E. Dolbear, of Tufts College, Mass., gave activity of every kind as the basis of all physiology. The body is an electro-magnetic machine, and electro-magnetic conditions regulate all its functions. These considerations give validity to a rational basis for electro-therapeutic study, and we may look forward with confidence to the time when all kinds of ailments will be intelligently met by electro-therapeutic practice.

"The Fundamental Relations of the Sinusoidal or Undulating to Other Forms of Current in their Action on Nervous

Tissue," by Walter Y. Cowl, M. D., of Berlin, Germany, was heard with interest.

L. Willard Reading, M. D., of Philadelphia, Pa., gave a paper on "Cases of Malignant Growth Treated by Electro-Puncture." He said that he has had great benefit result from electro-galvanic puncture in cases of malignant disease.

"Some Points in the Treatment of Stricture of the Urethra," by William Harvey King, M. D., of New York, attributed failure in the treatment of stricture of the urethra, to an improper knowledge of the anatomy and physiology of the stricture, also to improperly constructed instruments. Strictures are most common in the membranous portion of the urethra, just in front of which is a pouch where most of the instruments lodge and cannot be made to enter the stricture. This he proposes to remedy by making the staff of the sound larger to uniformly distend the canal, and to have the instrument correspond to the normal curve of the urethra.

"Electricity in Orificial Treatment," by C. A. Weirick, M. D., of Chicago, Ill., showed the benefit derived from the use of electricity in diseases of the orifices.

"Hypertrichosis and its Treatment by Electrolysis," by J. Coffin, M. D., of Boston, described the process of removing hairs. Dr. Coffin does not believe that their removal necessarily increases the growth of those remaining.

Dr. G. Gautier and T. Larat, of Paris, France, gave some interesting notes on "Electro-Therapeutics," showing that the local action, dependent upon nascent products, is superior in its effects to any other application.

George E. Percy, M. D., of Salem, Mass., gave a paper on "Treatment of Precidentia Uteri" by the faradic current.

Emily A. Bruce, M. D., of Boston, in "The Electrical Treatment of Dysmenorrhœa," said that she found both the faradic and galvanic current helpful in cases in conjunction with hygienic measures.

Jeannie W. Martine, M. D., of New York, gave "A New Method of Dilating with Faradism."

"Electricity as a Means of Diagnosis in Gynæcology," by

Dr. C. Apostoli, of Paris, France, explained the usefulness of electricity in three cases. The faradic current will relieve nervous and hysterical pain, but it is powerless where there is inflammation which is indicated by febrile reaction. The same study of the so-called galvanic reaction, informs us rapidly of the curability of these inflammatory lesions which the electric current has demonstrated, and, in consequence of this, tells us in one case to abstain from an operation, while in another it shows an operation to be urgent. Gynæcological Electro-Therapeutics, carefully and methodically applied, aids surgery instead of opposing it.

"Ovarian Cyst with Laparotomy" was the subject of a paper by M. Bonner Flinn, M. D., of Worcester, Mass.

"Reports of Gynæcological Cases," by Minnie C. T. Love, M. D., of Denver, Col., was the next paper.

"The Proper Application of the Electric Cautery for Nasal Diseases," by Wesley A. Dunn, M. D., of Chicago, Ill., gave the preference to electric cautery over other escharotics, as it is more under control of the operator and is followed by less reaction.

"Electricity in Diseases of the Pharynx," by Thomas L. Shearer, M. D., of Baltimore, Md., stated that in electricity the doctor finds a valuable remedy adjuvant in his practice, relieving cases that are intractable to other measures.

"The Use of Galvanism in Diseases of the Larynx," J. B. Garrison, M. D., of New York, said that in cases where there is a lack of nutrition of the laryngeal apparatus, much benefit is received by using the interrupted galvanic current applied over the inferior recurrent laryngeal nerve, stimulation of which increases the nutrition of the vocal apparatus.

Honorary membership was conferred by the Society upon Professor E. A. Dolbear, Tufts College, Mass.; Professor William L. Puffer, of Boston; Drs. G. Gautier and T. Larat, Paris, France; and Dr. J. Grand, Paris, France; also Professors Edwin J. Houston and A. E. Kennelly, Philadelphia, Pa.

The following officers were elected for the ensuing year: President, A. B. Norton, M. D., of New York; Vice-Presidents, F. A. Gardner, M. D., of Washington, D. C., and Clara

E. Gary, of Boston; Secretary, N. M. Frazer, of New York; Treasurer, J. B. Garrison, M. D., of New York; Executive Committee, William M. King, M. D., of New York, and William L. Jackson, M. D., of Boston.

It was unanimously voted that the meeting was the most interesting of any that has been held.

CLARA E. GARY, M. D.,
*Secretary.*

